# Copper amine oxidase 8 regulates arginine-dependent nitric oxide production in *Arabidopsis thaliana*

**DOI:** 10.1093/jxb/erx105

**Published:** 2017-04-05

**Authors:** Felicitas Groß, Eva-Esther Rudolf, Björn Thiele, Jörg Durner, Jeremy Astier

**Affiliations:** 1 Helmholtz Zentrum München, Department of Environmental Science, Institute of Biochemical Plant Pathology,D-85764 Neuherberg, Germany; 2 Forschungszentrum Jülich, Institute for Bio-and Geoscience, IBG-2, D-52428 Jülich, Germany; 3 Technical University Munich, Wissenschaftszentrum Weihenstephan, D-80333 München, Germany

**Keywords:** Arginase, arginine, copper amine oxidase, nitric oxide, nitric oxide production, nitrate reductase, plant.

## Abstract

Nitric oxide (NO) is a key signaling molecule in plants, regulating a wide range of physiological processes. However, its origin in plants remains unclear. It can be generated from nitrite through a reductive pathway, notably via the action of the nitrate reductase (NR), and evidence suggests an additional oxidative pathway, involving arginine. From an initial screen of potential *Arabidopsis thaliana* mutants impaired in NO production, we identified copper amine oxidase 8 (CuAO8). Two *cuao8* mutant lines displayed a decreased NO production in seedlings after elicitor treatment and salt stress. The NR-dependent pathway was not responsible for the impaired NO production as no change in NR activity was found in the mutants. However, total arginase activity was strongly increased in *cuao8* knockout mutants after salt stress. Moreover, NO production could be restored in the mutants by arginase inhibition or arginine addition. Furthermore, arginine supplementation reversed the root growth phenotype observed in the mutants. These results demonstrate that CuAO8 participates in NO production by influencing arginine availability through the modulation of arginase activity. The influence of CuAO8 on arginine-dependent NO synthesis suggests a new regulatory pathway for NO production in plants.

## Highlight

Copper amine oxidase in polyamine metabolism modulates arginine-dependent nitric oxide production by affecting arginase activity in *Arabidopsis thaliana*.

## Introduction

Nitric oxide (NO) is a ubiquitous radical gas that possesses a wide range of functions in plants. Indeed, this signaling molecule is involved in developmental processes, such as germination or flowering, as well as in the adaptive response to biotic or abiotic stresses (for reviews, see Scheler *et al.*, 2013; Yang *et al.*, 2014; Hichri *et al.*, 2015; Sanz *et al.*, 2015; Simontacchi *et al.*, 2015). Over the last years, the complex mechanisms underlying these effects have been studied in depth, including the NO-dependent post-translational modifications of proteins or the NO crosstalk with phytohormones (see, for example, Astier and Lindermayr, 2012; Freschi, 2013; Mur *et al.*, 2013).

However, the origins of NO in plants are not fully understood. In animals, NO is mainly produced via nitric oxide synthases (NOSs), which catalyze a two-step oxidation of l-arginine into l-citrulline and NO, using reduced NADPH as the electron donor, oxygen as co-substrate, and (6*R*-)-5,6,7,8-tetrahydrobiopterin (BH_4_), FAD, FMN, and calmodulin (CaM) as cofactors (Förstermann and Sessa, 2012).

In plants, the picture is more complex and the subject of debate (Moreau *et al.*, 2010; Frohlich and Durner, 2011). An extensive *in silico* study demonstrated that NOS homologs could not be found in the transcriptome of >1000 different photosynthetic organisms, with the notable exception of ~12 algae, including the recently characterized NOS from *Osterococcus tauri* (Foresi *et al.*, 2010; Jeandroz *et al.*, 2016). Importantly, no homologs were found in the transcriptomes of embryophytes (Jeandroz *et al.*, 2016).

In fact, two pathways for NO production have been described in plants. The reductive pathway converts nitrite to NO notably through the nitrite reductase activity (Ni-NR activity) of nitrate reductase (NR). The principal activity of NR concerns the reduction of nitrate to nitrite. However, it can also reduce nitrite into NO (Ni-NR activity) in an NADH-dependent reaction. This Ni-NR activity represents only ~1% of the total NR activity in normal conditions but can be promoted by several conditions such as acidic pH, anoxia, or a substantial increase in nitrite content (Rockel *et al.*, 2002; Meyer *et al.*, 2005). Although NR is the best characterized NO source in plants, other routes for nitrite reduction have also been described. Nitrite can be reduced to NO via the mitochondrial electron transport system (Gupta and Igamberdiev, 2016), or non-enzymatically in the case of high nitrite concentrations, low pH, or highly reducing conditions (Bethke *et al.*, 2004). Some evidence suggests that molybdoenzymes could also reduce nitrite in some conditions (Maia and Moura, 2015).

The second main pathway of NO production in plants is an oxidative one. Even if the enzymes involved in this pathway are unknown, several works have correlated consumption of l-arginine with a production of NO in plants, similar to the NOS activity present in animals (Gas *et al.*, 2009; Del Río, 2011; Corpas and Barroso, 2014). This NOS-like activity is further supported by the expression of recombinant rat NOS in *Arabidopsis thaliana* and *Nicotiana tabacum*, which results in increased NO production and higher resistance to biotic and abiotic stresses (Chun *et al.*, 2012; Shi *et al.*, 2012). In addition, several other experimental data support the existence of an oxidative route for NO production in plants, such as external addition of hydroxylamine or polyamines (PAs), which both produce NO in plant cells (Tun *et al.*, 2006; Rümer *et al.*, 2009; Yang *et al.*, 2014; Jeandroz *et al.*, 2016).

Besides these works directly linking the NO produced in plants to a putative NOS activity, several indirect reports also favor the existence of arginine-dependent NO production in plants. For example, an increased arginase activity, an enzyme directly modulating the arginine and PA bioavailability in the plant, resulted in an impaired NO production in *A. thaliana* correlated with higher salt stress resistance (Flores *et al.*, 2008; Shi *et al.*, 2013; Meng *et al.*, 2015). Moreover, copper amine oxidase 1 (CuAO1) mutants displayed decreased NO production in *A. thaliana* after abscisic acid (ABA) or PA treatment (Wimalasekera *et al.*, 2011). CuAOs are homodimeric enzymes that control PA metabolism by catalyzing the oxidation of the primary amine groups of PAs, with a higher affinity for putrescine and cadaverine (Cona *et al.*, 2006). The catabolic products from these oxidation reactions are also involved in signaling and regulation of primary metabolism. As an example, the 4-aminobutanal produced by CuAO from putrescine is a precursor of γ-aminobutyric acid (GABA), a well-known signaling compound involved in plant stress responses (Moschou *et al.*, 2012). To date, 10 different putative CuAOs have been annotated in *A. thaliana*, four of them (AtAO1, AtCuAO1, AtCuAO2, and AtCuAO3) being recently characterized (Wimalasekera *et al.*, 2011; Planas-Portell *et al.*, 2013; Ghuge *et al.*, 2015).

In this study, CuAO8 was identified by a targeted screen to be involved in the regulation of NO production. We were able to show that CuAO8 participates in NO production after 2,6-dichloroisonicotinic acid (INA) and salt stress, but also impacts primary root growth. We demonstrated that CuAO8, a true copper amine oxidase, modulates NO production by regulating arginase activity, thereby affecting the bioavailability of arginine.

## Materials and methods

### Plant growth

All *A. thaliana* T-DNA insertion lines were in the Columbia (Col-0) background and grown vertically in square Petri dishes on half-strength Murashige and Skoog (1/2 MS) medium [1% sucrose, 1.2% phytoagar (Duchefa)] in short-day conditions (10 h light d^–1^) unless indicated otherwise. Seeds were surface sterilized for 3 h with chlorine gas and vernalized for 2 d at 4 °C in the dark.

### T-DNA insertional knockout lines

T-DNA insertion lines used were tested for homozygosity by PCR on genomic DNA using gene-specific primers in combination with the LBb.1.3 T-DNA insertion primer. The primer sequences were obtained from the SALK Institute with the iSect Primers tool: http://signal.salk.edu/tdnaprimers.2.html, last accessed 24 February 2017 (not shown). For knockout verification of *cuao8-1* and *cuao8-2*, total RNA was isolated (Qiagen), cDNA was synthesized (Qiagen), and CDS (coding sequence) transcript absence checked by RT-PCR using *CuAO8*-specific primers (see Supplementary Table S1 at *JXB* online).

### Relative quantitative PCR

RNA (Qiagen) was isolated from 5-day-old seedlings harvested before or after NaCl (200 mM/6 h) treatment. Afterwards cDNA was synthesized (QuantiTect Reverse transcriptase kit, Qiagen) according to the manufacturer’s instructions. Primers (Supplementary Table S1) were designed using QuantPrime software discriminating splice variants and span exon–exon borders (http://quantprime.mpimp-golm.mpg.de/, last accessed 24 February 2017) (Arvidsson *et al.*, 2008). The qPCR was performed with Applied Biosystems 7500 (Fast) and the Sequence Detection Software 1.3.1 from Applied Biosystems. Raw data analysis and calculation of the efficiency (*E*) and cycle threshold (CT) values were carried out with the PCR-Miner software http://ewindup.info/miner/, last accessed 24 February 2017 (Zhao and Fernald, 2005). The relative transcript abundance was calculated as (1+*E*)^−CT^ for every well and normalized against the geometric mean of the reference genes ubiquitin 5, tubulin 9, and ribosomal protein S16 (Scheler *et al.*, 2015; Supplementary Table S1).

### Total protein extraction for CuAO8 knockout analysis

Total protein of 4-week-old leaves was extracted using the Plant Total Protein Extraction Kit (Sigma-Aldrich) according to the manufacturer’s instructions.

### Quantification of NO and H_2_O_2_ production

Five-day-old seedlings were stained with diverse dyes reflecting the NO/H_2_O_2_ production during stress treatments. Different dyes were used in different concentrations and for different incubation times: DAF-FM DA (4-amino-5-methylamino-2',7'-difluorofluorescein diacetate; 15 µM/15 min; Sigma-Aldrich), DAR-4M AM (diaminorhodamine; 5 µM/60 min; Sigma-Aldrich); Cu_2_(FL2E) (5 µM/60 min; STREM Chemicals); Amplex Red (100 µM/20 min; Invitrogen); and DCF-DA (2′,7′-dichlorofluorescein diacetate; 20 µM/15 min; Sigma-Aldrich). All stainings and treatments were performed in STM buffer (50 mM MES-KOH pH 5.7, 0.25 mM KCl, 1 mM CaCl_2_). After staining, the seedlings were washed three times in STM buffer and treated with 2 mM INA (Sigma-Aldrich) for 45 min or 200/150 mM NaCl for 6/5.5 h, respectively. INA is an analog of salicylic acid, resulting in a strong NO production when perceived in plants (Floryszak-Wieczorek *et al.*, 2012). cPTIO [2-(4-carboxyphenyl)-4,4,5,5-tetramethylimidazoline-1-oxyl-3-oxide; 200 µM; Sigma-Aldrich], l-arginine (1 mM; Sigma-Aldrich), or *nor*-NOHA (*N* ω-hydroxy-nor-arginine; 0.1 mM; Sigma-Aldrich) were applied during both the staining and the stress application phase. The roots were observed under an epifluorescence microscope (Olympus BX61, ×4 objective) with enhanced green fluorescent protein (eGFP; excitation filter, 474/23; emission filter, 525/45) or red (excitation filter, 585/20; emission filter, 647/57) filter settings. The microscope software (cellP/cellSens, Olympus Soft Imaging) was set on optimized histogram and flexible exposure time for single optimized images. The microscope image each time included both the tested sample and a stress-treated Col-0 sample as internal control. The fluorescence intensity of the sample was further quantified relative to this internal control using ImageJ software. Afterwards, the values of the relative quantification were normalized to the genotype-specific non-treated control.

### Primary root length measurements

Plants were grown on vertical 1/2 MS plates (1% sucrose, 1.2% phytoagar) supplemented with either l-arginine (1 mM), GABA (1 mM), GSNO (*S*-nitrosoglutathione; 50 µM), or SNAP (*S*-nitroso-*N*-acetylpenicillamine; 50 µM) (Sigma-Aldrich). After vernalization, they were subjected to long-day conditions (14 h light d^–1^) for 11 d and scanned. The primary root length was determined using the software ImageJ.

### Transient protein expression and purification of CuAO8-His_6_

For transient expression of CuAO8-His_6_, the modular transfection system magnICON^*®*^ (Bayer CropScience GmbH, Marillonnet *et al.*, 2005) was used (3'-provector module pICH11599 carrying CuAO8-His_6_; integrase module pICH14011; 5'-provector module pICH17388). *CuAO8* gene sequence (GenBank accession no. NM_102904) was amplified with *CuAO8*-specific primers (Supplementary Table S1) from cDNA synthesized from 4-week-old *A. thaliana* leaves (Quiagen kits). All constructs were verified by sequencing. All three vectors were separately transformed in *A. tumefaciens* GV3101 pMP90, and further grown in selective LB medium at 28 °C for ~24 h. The bacteria were harvested by centrifugation (4000 rpm, 10 min), and washed twice with infiltration buffer (10 mM MES-KOH pH 5.7, 10 mM MgCl_2_). The pellets were resuspended in infiltration buffer (OD_600_=0.3). The cultures were incubated for 3 h at room temperature and afterwards mixed in a 1:1:1 ratio. This mixture was infiltrated with a syringe in the abaxial side of 6-week-old *Nicotiana benthamiana* leaves. At 10 days after infiltration (dpi), the leaves were harvested and further ground in liquid nitrogen. Two volumes of extraction buffer were added [1× phosphate-buffered saline (PBS), 150 mM NaCl, 10% glycerin, 1% Triton X-100, 1 mM DTT, 1× protease inhibitor cocktail (cOmplete, EDTA free, Roche)] and the suspension incubated for 15 min on ice, with regular vortexing. After centrifugation (14 000 rpm, 25 min, 4 °C), the supernatant was rebuffered using ZebaSpin desalting columns (Thermo Fisher Scientific) [1× PBS, 150 mM NaCl, 10% glycerin, 0.1% Triton X-100, 1 mM DTT, 1× protease inhibitor cocktail (Roche)]. The rebuffered extract was loaded on a pre-equilibrated Ni-NTA column (Qiagen). The column was washed using 30 column volumes (CVs) of desalting buffer followed by 8 CVs of desalting buffer containing 50 mM imidazole. Elution was performed with 4 CVs of desalting buffer containing 100, 200, or 300 mM imidazole. The eluted fractions were concentrated and rebuffered in 1× PBS with 10K Amicon Ultra-4 (Merck, Germany) filter units. The protein concentration was measured with Bradford reagent (Bio-Rad), and the presence of CuAO8 was verified by SDS–PAGE and by western blotting with anti-His-tag antibody (ab137839, Abcam) and/or anti-CuAO8 antibody (Core Facility Monoclonal Antibody Development, Helmholtz-Zentrum München, Germany).

### In vitro *CuAO8 activity measurement*

The H_2_O_2_ production by CuAO8 was measured with the Amplex^®^ Red Hydrogen Peroxide/Peroxidase Assay Kit (Planas-Portell *et al.*, 2013) (ThermoFisher Scientific, No. A22118) according to the manufacturer’s instructions. Briefly, 100 ng of CuAO8 was incubated with 1 mM of substrate (Sigma-Aldrich) in a total reaction volume of 100 µl. H_2_O_2_ production was detected using resorufin (detecting in a 1:1 ratio) at the specific excitation and emission wavelengths (excitation, 571 nm; emission, 585 nm) with the TECAN Reader Infinite M1000pro spectrophotometer using a black 96-well plate. The amine oxidase inhibitor aminoguanidine (0.1 mM, Sigma-Aldrich) was used in combination with the provided substrates. In parallel, NO-producing activity was assayed in the same conditions replacing the Amplex Red probe with DAR-4AM DA (excitation, 543 nm; emission, 575 nm).

### Determination of polyamines

The levels of free putrescine, spermidine, and spermine in 5-day-old seedlings were quantified with HPLC after pre-column derivatization with FMOC-Cl (Fellenberg *et al.*, 2012). A 100 mg aliquot of plant material was ground in liquid nitrogen and extracted with 1 ml of 5% perchloric acid for 1 h at room temperature in the dark with frequent vortexing. After centrifugation (18 000 *g*, 10 min), 15 µl of the supernatant was neutralized with 360 µl of 0.1 M NaHCO_3_ supplemented with 1,7-diaminoheptane as internal standard. After the addition of 100 µl of acetone and 200 µl of 6 mM FMOC, the samples were incubated for 5 min at room temperature and for 10 min at 50 °C. The reaction was stopped at –20 °C for 5 min and 300 µl of methanol were added. The derivatized PAs were separated by reverse phase chromatography on a Luna C18 column [5 µm 100 Å C18(2) 250 × 4.6 mm column, Phenomenex] connected to a Beckman System Gold HPLC equipped with a Shimadzu RF 10AxL fluorescence detector (excitation, 260 nm; emission, 313 nm). The flow rate and the column temperature were set at 1 ml min^–1^ and 20 °C. Elution was performed with water as eluent A and methanol as eluent B. Elution conditions were (min/B%): 0/80; 30/100, 36/100, 42/80, and 45/80. Quantification was based on the external standard method using calibration curves fitted by linear regression analysis in combination with the internal standard method (standards: putrescine dihydrochloride, 1,7-diaminoheptane, spermidine, and spermine), where the response factor of each derivative was corrected with respect to that of the internal standard.

### Measurement of nitrate reductase activity

The NR activity was measured as the rate of nitrite production determined with a spectrophotometric assay (Frungillo *et al.*, 2014). All samples were protected from light. Total protein from seedlings was extracted in 50 mM HEPES-KOH pH 7.5, 0.5 mM EDTA, 100 µM FAD, 5 mM Na_2_MoO_4_, 6 mM MgCl_2_, protease inhibitor cocktail (Roche), and the homogenate was incubated for 10–15 min on ice with periodic vortexing. After centrifugation, the supernatant was rebuffered using 10K Amicon Ultra 0.5 centrifugal filter units (Millipore, No. UFC501008) and the protein concentration was measured with Bradford reagent. The protein activity assay was performed in a clear 96-well plate (Greiner) in a total reaction volume of 200 µl. The reaction mixture consisted of 1 mM KNO_3_, 1 mM NADH, and either 2 mM EDTA or 6 mM MgCl_2_, and was incubated for 55 min at room temperature with gentle shaking. Then, a 1:1 mixture of 1% (w/v) sulfanilamide in 1.5 M HCl and 0.02% (w/v) *N*-(1-naphthyl)-ethylenediamine dihydrochloride in 1.5 M HCl was added. After 15 min incubation at room temperature, the *A*_540_ was measured with the TECAN Reader Infinite M1000pro spectrophotometer. The activity in the presence of EDTA represents the total NR activity and the activity, measured in the presence of MgCl_2_ represents the actual NR activity.

### Measurement of nitrite and nitrate content

The nitrite and nitrate contents in 5-day-old seedlings were determined with the Nitric Oxide Analyzer Sievers 280i from GE Healthcare. A 500 µl aliquot of 1× PBS was added to 200 mg of seedlings ground in liquid nitrogen and incubated on ice for 10 min with periodic vortexing. After centrifugation (12 000 rpm, 10 min, 4 °C), the supernatant was used for analysis. For nitrite detection, NO_2_^–^ was reduced to NO by I_3_^–^ (triiodide, 30 °C). For nitrate detection, nitrite and nitrate were reduced using VCl_3_ (vanadium chloride, 90 °C) and the measured amount of nitrite was subtracted. The nitrite/nitrate content was normalized to the protein content in the supernatant measured with Bradford reagent.

### Measurement of arginase activity

To measure arginase activity in total protein extracts of 5-day-old seedlings, the Arginase Activity Assay Kit (Sigma-Aldrich, No. MAK112) was used according to the manufacturer’s instructions. Total protein extracts were homogenized in 50 mM Tris–HCl pH 9 and the supernatant was rebuffered using a 10K Amicon Ultra 0.5 centrifugal filter (Millipore). The protein concentrations were measured with Bradford reagent (BioRad).

### Amino acid analysis

Proteinogenic amino acids were measured according to Thiele *et al.* (2008) with modifications. The extraction was conducted from 100 mg of 5-day-old seedlings homogenized with an aqueous HCl–ethanol mixture containing an internal standard (d_5_-Phe). After incubation on ice and centrifugations, the supernatant was analyzed by liquid chromatography electrospray ionization–tandem mass spectrometry technique (LC-ESI-MS-MS) in the positive electrospray ionization mode. Analyses were conducted with a Waters ACQUITY UPLC system (binary pump, autosampler) coupled to a Waters Xevo TQ-S triple–quadrupole mass spectrometer (Waters Technologies Corp., MA, USA). A 10 µl extract was injected into the UPLC system. Amino acids were separated on a Nucleosil 100-5 SA column (150 × 2 mm, 5 μm particle size) equipped with a pre-column filter from Macherey-Nagel (Düren, Germany). The mobile phase consisted of 5% acetic acid (A) and 30 mM ammonium acetate (pH 6, B). Samples were separated at 40 °C and a flow rate of 500 µl min^–1^ using two consecutive isocratic steps: isocratic at 87.5% A for 15 min, linear gradient to 0% A over 0.5 min, isocratic at 0% A for 4.5 min, linear gradient to 87.5% A over 0.5 min, and equilibration at 87.5% A for 9.5 min. The capillary voltage was set to 2.5 kV. The cone voltage was 20 V. The dissolvation temperature was 500 °C and source temperature 150 °C. The dissolvation gas flow was set to 800 l h^–1^, the cone gas flow was set at 150 l h^–1^ using nitrogen in both cases. MRM was used for specific quantification of the amino acids and the internal standard, applying a dwell time of 0.018 s. Nitrogen was used as the collision gas at a flow rate of 0.25 ml min^–1^. MS-MS parameters of the amino acids were determined by flow injection analysis of amino acid standard solutions using the inbuilt syringe pump (Supplementary Table S3).

### Transient expression and localization of GFP–CuAO8 in *N. benthamiana*

GFP–CuAO8 was obtained using the Gateway recombination system (Invitrogen) with the pENTR/D-TOPO entry vector and the pK7WGF2 expression vector (adding an N-terminal eGFP). The CuAO8 gene sequence was amplified with specific primers (Supplementary Table S1) applied on cDNA synthesized from 4-week-old *A. thaliana* leaves. *Agrobacterium tumefaciens* GV3101 pmP90 was transformed either with pK7WGF2:CuAO8 or with p19 RNA silencing suppressor vector. The agrobacteria were grown in selective LB medium at 28 °C until an OD_600_ of 1.7–2.2. After harvesting by centrifugation (4500 rpm, 10 min), the pellet was washed twice with buffer (10 mM MES-KOH pH 5.7, 10 mM MgCl_2_), resuspended to an OD_600_ of 1.3, and incubated for 3 h at room temperature. Then suspensions were mixed in a 1:1 ratio and infiltrated with a syringe in the abaxial side of 5-week-old *N. benthaniama* leaves. A 5 dpi, the GFP expression was monitored with a confocal microscope (Zeiss 510 META, C-Apchromat ×40/1.2 water objective). Leaf discs were vacuum infiltrated three times in FM4-64 (Biotium) solution (20 µM) followed by a 15 min incubation in the dark to visualize the plasma membrane (excitation/emission filter: GFP, 488 nm/BP 505–530; FM4-64, 514 and 543/BP 565–615 IR).

## Results

### The mutant *cuao8-1* displays an impaired NO production after elicitor treatment in seedling root tips

**Fig. 1.**
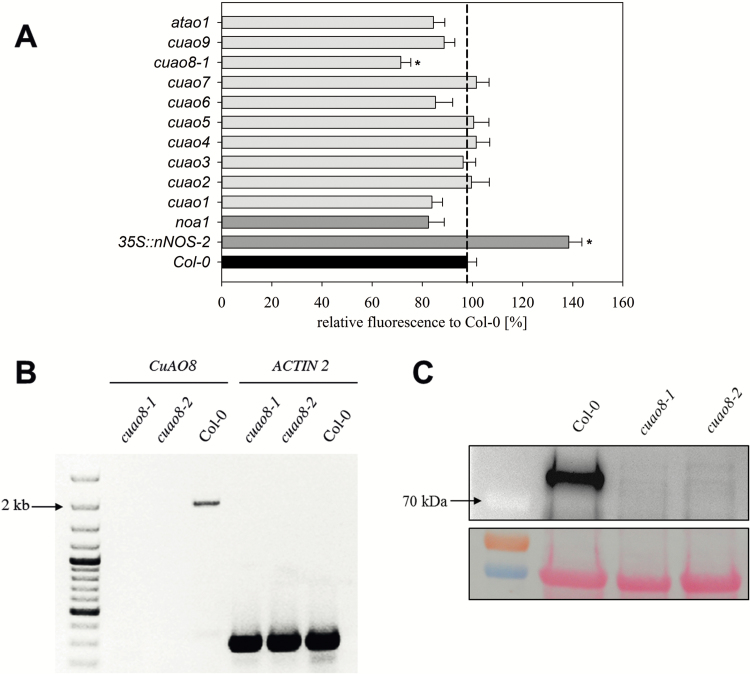
Screen of INA-induced NO production in root tips of different *A. thaliana* mutant lines and verification of the complete knockout of *CuAO8* in *cuao8-1* and *cuao8-2* T-DNA insertion lines on the mRNA and protein level. (A) Five-day-old seedlings were stained with DAF-FM DA (15 µM, 15 min) and then treated with INA (2 mM, 45 min), to induce NO production. The resulting fluorescent signal was observed with an epifluorescence microscope and quantified using ImageJ. The fluorescent signal of the mutant line was normalized relative to Col-0 set as 100%. Means ±SE of at least three individual experiments are shown (*n*=19–31). *Significant difference *P*<0.05 based on ANOVA on ranks (Dunn’s test) with respect to Col-0. (B) Transcriptional analysis of *CuAO8*: the expression of the *CuAO8* gene was tested in 4-week-old leaves. As a loading control, *ACTIN 2* was amplified. (C) Western blot analysis of CuAO8 on protein extracts from Col-0, *cuao8-1*, and *cuao8-2* plants. Total protein extracts of 4-week-old leaves were separated by SDS–PAGE and western blotted. The membrane was probed with anti-CuAO8 primary antibody and a secondary antibody coupled with horseradish peroxidase (HRP). Loading control: Ponceau staining. (This figure is available in colour at *JXB* online.)

We performed an initial screening experiment to monitor the NO production in several CuAO mutant lines of *A. thaliana* (Planas-Portell *et al.*, 2013; Supplementary Table S2). The *noa1* (*nitric oxide associated 1*) mutant, which is impaired in NO production (Guo *et al.*, 2003), and an overexpression line of the rat neuronal nitric oxide synthase 35S::nNOS-2 (Shi *et al.*, 2012) were included as negative and positive controls, respectively (Fig. 1A). NO production was triggered with INA treatment in root tips of 5-day-old seedlings and estimated using the fluorescent probe DAF-FM DA, which reacts with intracellular NO and emits fluorescence (Kojima *et al.*, 1998).

Of the 13 lines tested, the lines *noa1*, *atao1* (*aldehyde oxidase 1*), *cuao1*, *cuao6*, *cuao8*, and *cuao9* displayed a reduced NO production, even if the difference was not significant. The *cuao1* and *noa1* lines presented a reduced signal, in agreement with the previously published data (Guo *et al.*, 2003; Wimalasekera *et al.*, 2011). Interestingly, only the line impaired in the expression of the putative *CuAO8* showed a significantly decreased fluorescence after INA treatment (Fig. 1A). In contrast, the 35S::nNOS-2 overexpressor showed a drastically increased NO production, in accordance with expectations.

**Fig. 2.**
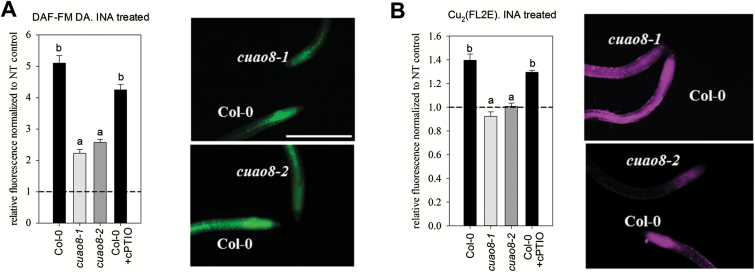
INA-induced NO production in Col-0, *cuao8-1*, and *cuao8-2* root tips. Fluorescence quantification analysis and representative images (scale bar=500 µm) of 5-day-old root tips from Col-0, *cuao8-1*, and *cuao8-2* stained with DAF-FM DA (A, 15 µM, 15 min) or Cu_2_(FL2E) (B, 5 µM, 45 min) and treated with INA (2 mM, 45 min). The fluorescent signal in the root tips was observed with an epifluorescence microscope. Shown is the relative fluorescence normalized to non-treated controls of each genotype. The NO scavenger cPTIO (200 µM) was added during staining and treatment. Means ±SE of at least three individual experiments are shown [DAF-FM DA, *n*=24–34; Cu_2_(FL2E), *n*=21–29]. Different letters indicate a statistically significant difference based on ANOVA on ranks (Dunn’s test. *P*<0.05).

To confirm these preliminary results, two independent *cuao8* knockout lines (Supplementary Table S2) were confirmed at the transcriptomic (Fig. 1B) and the proteomic level (Fig. 1C). We further checked the NO production capacities of these lines after INA treatment, using DAF-FM DA (Fig. 2A) and the more selective NO probe Cu_2_(FL2E) (McQuade and Lippard, 2010; Fig. 2B). Both mutant lines displayed a strong reduction of NO production after INA stress, confirming the results obtained in the initial screen. As a control, a Col-0 induced sample was co-treated with the NO scavenger cPTIO, and showed non-significantly reduced NO production. These results demonstrate the existence of a CuAO8-dependent NO production in seedling root tips after elicitor treatment.

### *cuao8-1* and *cuao8-2* are impaired in NO production after salt stress

**Fig. 3.**
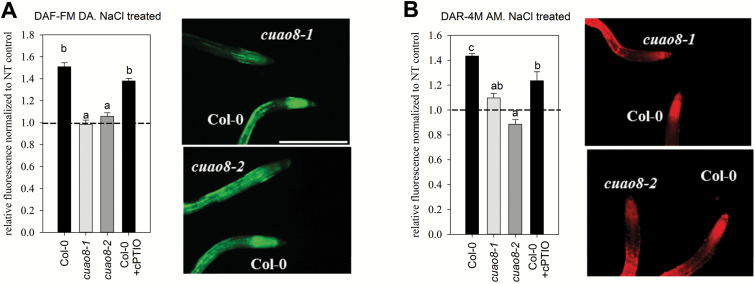
NaCl-induced NO production in Col-0, *cuao8-1*, and *cuao8-2* root tips. Fluorescence quantification analysis and representative images (scale bar=500 µm) of 5-day-old root tips from Col-0, *cuao8-1*, and *cuao8-2* stained with DAF-FM DA (A, 15 µM, 15 min) or DAR-4M AM (B, 5 µM, 60 min) and treated with NaCl (DAF-FM DA staining, 150 mM, 5.5 h; DAR-4M AM staining, 200 mM, 6 h). The fluorescent signal in the root tips was observed with an epifluorescence microscope. Shown is the relative fluorescence normalized to non-treated controls of each genotype. The NO scavenger cPTIO (200 µM) was added during staining and treatment. The means ±SE of at least three individual experiments are shown (DAF-FM DA, *n*=22–27; DAR-4M AM, *n* = 24–34). Different letters indicate a statistically significant difference based on ANOVA on ranks (Dunn’s test. *P*<0.05).

To determine the role of CuAO8 in NO production, we subjected *cuao8-1* and *cuao8-2* seedlings to salt stress. Both *cuao8* mutants displayed an impaired NO production in root tips after salt stress, verified with the two different fluorescent probes DAF-FM DA (Fig. 3A) and DAR-4M AM (Fig. 3B). Remarkably, no induction of NO production was measured after salt stress in the mutants, as compared with the wild type.

These results suggest the existence of a general CuAO8-dependent NO production in seedlings root tips of *A. thaliana*.

### CuAO8 impacts primary root length

**Fig. 4.**
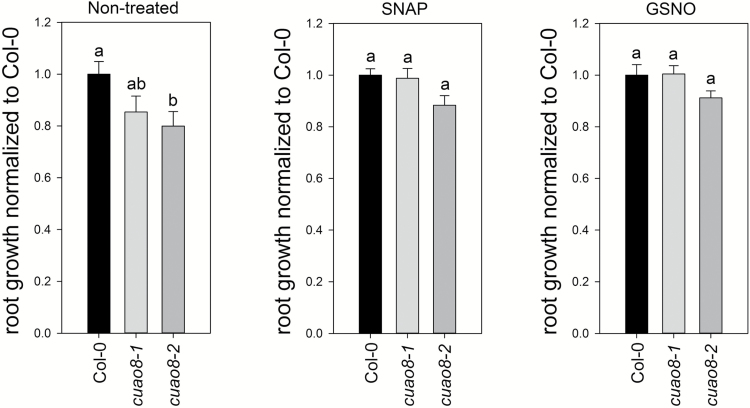
Relative root growth of Col-0, *cuao8-1*, and *cuao8-2* after 11 d on vertical 1/2 MS plates (Non-treated) or 1/2 MS plates supplemented with GSNO (50 µM) or SNAP (50 µM). Means ±SE of at least three individual experiments are shown (*n*=14–22), normalized to Col-0. Different letters indicate a statistically significant difference based on ANOVA on ranks (Dunn’s test. *P*<0.05).

We further analyzed the phenotype of the *cuao8* mutants looking for primary root length of seedlings grown of 1/2 MS medium (Fig. 4). Mutants displayed 15% and 30% shorter primary root length, respectively, as compared with Col-0, to a significant extent for the *cuao8-2* line. Interestingly, the inclusion of NO donors (GSNO and SNAP) in the medium could restore the primary root length to the Col-0 level (Fig. 4). These results demonstrate that the CuAO8-dependent NO production is involved in controlling the primary root growth of seedlings.

### CuAO8 possesses a typical copper amine oxidase activity

**Fig. 5.**
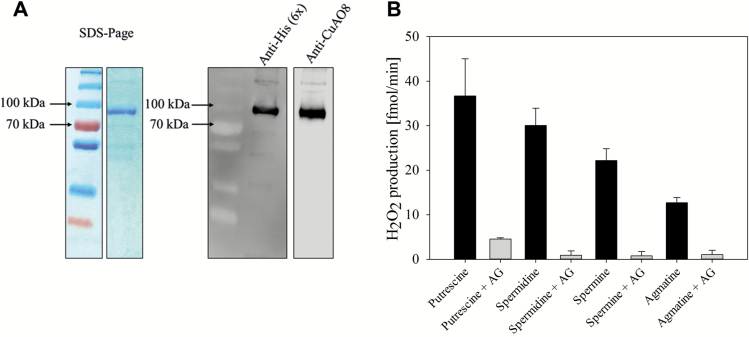
Purification and activity test of recombinant CuAO8. (A) SDS–PAGE and western blot of recombinant CuAO8 with a C-terminal His-tag (6×), transiently expressed in *N. benthamiana.* Shown are representative SDS–PAGE (Coomassie staining) and western blot of CuAO8 of the Ni-NTA elution fraction (200 mM imidazole). The western blot was probed with primary anti-His or anti-CuAO8 antibody and a secondary antibody coupled with HRP. (B) Amine oxidase activity was tested with the Amplex Red peroxidase test detecting H_2_O_2_ produced by CuAO8-His_6_. A 100 ng aliquot of protein was incubated with 1 mM of substrate alone or in combination with 0.5 mM of an amine oxidase inhibitor (AG, aminoguanidine). The means ±SE of three individual experiments are shown. (This figure is available in colour at *JXB* online.)

To characterize further the function of CuAO8, we transiently expressed the recombinant protein in *N. benthamiana* leaves. Recombinant CuAO8-His_6_ was obtained after an Ni-NTA column purification as shown by SDS–PAGE and verified by western blot analysis applying anti-His and anti-CuAO8 antibody (Fig. 5A). The band appeared at 95 kDa, which is higher than predicted, but glycosylation events, as already shown in apples and human amine oxidases, are likely to increase the size of the protein (Airenne *et al.*, 2005; Zarei *et al.*, 2015).

The recombinant CuAO8 displayed no NO production activity (data not shown). The enzymatic activity of CuAO8 was further examined *in vitro* by measuring the amount of H_2_O_2_ released from the oxidative deamination of PAs. H_2_O_2_ was detected using the Amplex Red reagent as previously described (Planas-Portell *et al.*, 2013). The results showed that CuAO8 possesses a typical CuAO activity, with the highest activity for putrescine as a substrate, and to a lower extent for spermidine, spermine, and agmatine (Fig. 5B). This activity was almost completely abolished with co-application of the irreversible inhibitor of amine oxidases aminoguanidine (AG), implying specific CuAO activity of CuAO8. These results demonstrate that CuAO8 is a typical copper amine oxidase.

### H_2_O_2_ production and polyamine accumulation in *cuao8* mutants

**Fig. 6.**
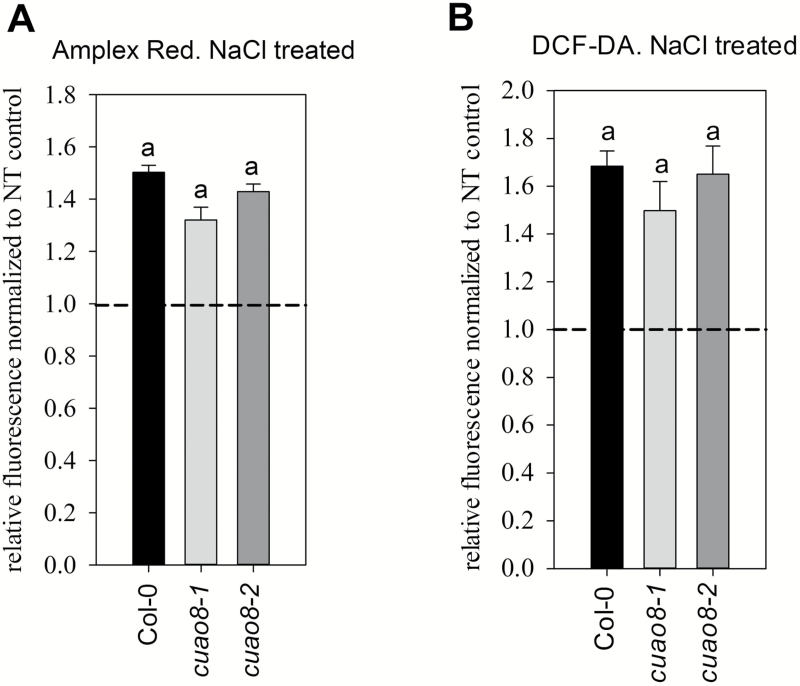
NaCl-induced H_2_O_2_ production in Col-0, *cuao8-1*, and *cuao8-2* root tips. Fluorescence quantification analysis of 5-day-old seedlings from Col-0, *cuao8-1*, and *cuao8-2* stained with Amplex Red (A) or DCF-DA (B) and treated with NaCl (200 mM, 6 h). The fluorescent signal in the root tips was observed with an epifluorescence microscope. Shown is the relative fluorescence normalized to non-treated controls of each genotype. Means ±SE of at least three individual experiments are shown (Amplex Red, *n*=22–30; DCF-DA, *n*=21–28). Different letters indicate a statistically significant difference based on ANOVA on ranks (Dunn’s test. *P*<0.05).

Since CuAO8 enzymatic activity results in the production of H_2_O_2_, the *in vivo* generation of this compound was followed in root tips of seedlings from Col-0 and mutant lines after salt stress exposure using Amplex Red reagent and the fluorescent dye DCF-DA. Only a minor and non-significant reduction of H_2_O_2_ production was measured in *cuao8* mutants (Fig. 6). Although H_2_O_2_ is able to induce NO production in seedlings of *A. thaliana* (Wang *et al.*, 2010), this suggests that the minor reduction of H_2_O_2_ production cannot explain the reduction in NO production observed in *cuao8-1* and *cuao8-2*.

We then determined the different PA contents of Col-0 and mutant lines in control and salt-treated seedlings by HPLC (Table 1). Without stress, no significant differences were observed between Col-0, *cuao8-1*, and *cuao8-2* for any of the PA contents. Interestingly, after salt stress, a higher amount of putrescine was detected in mutant lines as compared with Col-0, significantly for *cuao8-2*. This demonstrates that CuAO8 deaminates putrescine in response to salt stress in *A. thaliana*.

**Table 1.** Free polyamine levels (nmol g FW^–1^) in 5-day-old seedlings of Col-0, *cuao8-1*, and *cuao8-2* after NaCl (200 mM, 6 h) or control (buffer, 6 h) treatment

		**Col-0**	***cuao8-1***	***cuao8-2***
**Putrescine**	Control	41.60 ± 3.55	49.41 ± 2.76	44.25 ± 8.43
	NaCl	31.01 ± 1.95	49.55 ± 9.55	51.08 ± 5.65*
**Spermidine**	Control	313.37 ± 3.24	282.58 ± 12.67	239.57 ± 4.05
	NaCl	264.45 ± 57.93	294.98 ± 12.78	248.50 ± 15.33
**Spermine**	Control	23.65 ± 1.11	21.76 ± 0.89	20.70 ± 1.55
	NaCl	20.33 ± 4.56	23.92 ± 1.75	22.51 ± 1.29

Means ±SE of three individual experiments are shown.

*Significant difference *P*<0.05 based on *t*-test with respect to the corresponding Col-0.

These results support the activity tests done *in vitro* and argue in favor of a typical CuAO activity for CuAO8 *in vivo* being involved in the salt stress response in seedling root tips.

### Nitrate reductase activity is not impaired in *cuao8* mutant lines

As we revealed a typical CuAO activity for CuAO8, we investigated the possible reason for the impairment of NO production observed after elicitor and salt treatment in *cuao8-1* and *cuao8-2*.

**Fig. 7.**
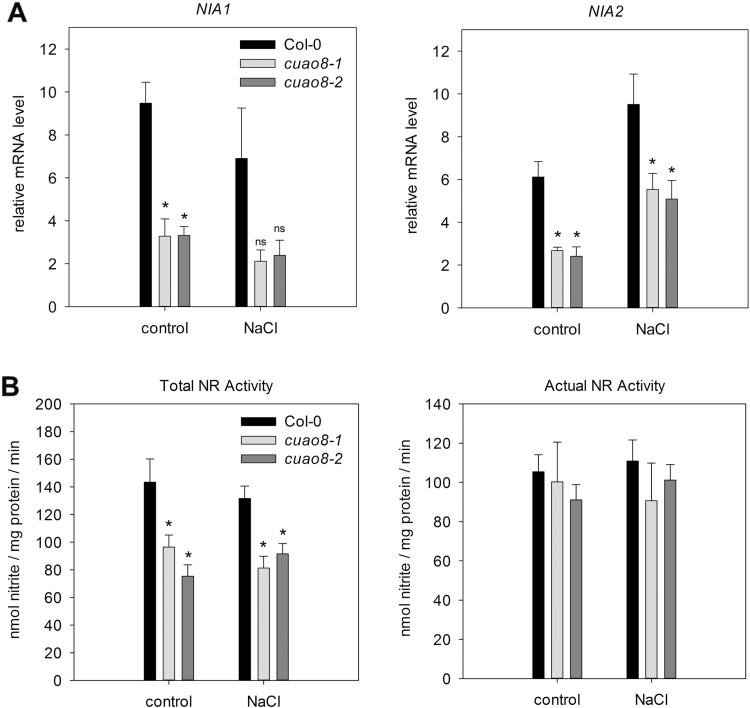
Relative mRNA level of *NIA1* and *NIA2* and nitrate reductase activity test of 5-day-old seedlings of Col-0, *cuao8-1*, and *cuao8-2* after NaCl treatment. (A) Relative expression of nitrate reductase 1 (*NIA1*) and 2 (*NIA2*) in 5-day-old seedlings after control (buffer, 6 h) or NaCl treatment (200 mM, 6 h). (B) Five-day-old seedlings were treated with NaCl (200 mM, 6 h) or buffer (control, 6 h) and NR activity was measured in total protein extracts. NR activity was determined in the presence of EDTA (total NR activity) or MgCl_2_ (actual NR activity). The means ±SE of at least three individual experiments are shown. *Significant difference *P*<0.05 based on ANOVA (Holm–Sidak test) with respect to Col-0.

To investigate the role of NR after salt stress in the *cuao8* mutants, we followed by qPCR the expression of the two genes encoding nitrate reductases in *A. thaliana*, namely *NIA1* and *NIA2* (Fig. 7A). The expression of these genes followed a similar pattern in the different lines tested, *NIA1* expression being stable and *NIA2* being induced after salt stress compared with the control (Fig. 7A). However, *NIA1* and *NIA2* gene expression was reduced in *cuao8-1* and *cuao8-2*, both with and without salt stress.

To test whether this down-regulation was related to a change in the enzymatic activity, the NR activity was then determined in the Col-0 and mutant lines, submitted or not to salt stress (Fig. 7B). Since NR activity is tightly regulated at the post-translational level by phosphorylation, subsequent 14-3-3 protein binding, and inhibition in an Mg^2+^-dependent manner (Kaiser and Huber, 2001; Heidari *et al.*, 2011), we assayed NR activity in the presence of EDTA (total NR activity) or excess Mg^2+^ (actual NR activity). The total NR activity was found to be significantly reduced in the two mutant lines, which fits with the qPCR results obtained (Fig. 7B). However, the actual NR activity was similar in Col-0, *cuao8-1*, and *cuao8-2*, which suggests that NR activity is not the reason for the impaired NO production observed in the mutants. This result shows that despite a modification of the expression of NR genes, the NR activity itself is not responsible for the decreased NO production observed in *cuao8* mutant lines after salt stress.

The reductive NO-producing pathway from nitrite depends on the nitrate/nitrite content and is promoted by high nitrite concentrations. To test whether the decreased NO production in the mutant lines was due to decreased nitrite concentrations, we determined the amount of nitrite and nitrate in the seedlings (Table 2). The impaired NO production of CuAO8 mutants did not correlate with a decreased nitrite availability, as no significant differences were found in mutant nitrite contents as compared with Col-0. In contrast, higher but not significant amounts of nitrite were monitored in these lines. A similar trend was observed for nitrate contents. These results exclude any implication of the nitrite/nitrate content in the impairment of NO production observed in the mutant lines.

**Table 2.** Nitrate and nitrite quantification in 5-day-old seedlings of Col-0, *cuao8-1*, and *cuao8-2* after NaCl (200 mM, 6 h) or control (buffer, 6 h) treatment

		**Col-0**	***cuao8-1***	***cuao8-2***
Nitrite (pmol mg protein^–1^)	Control	146.3 ± 27.8	350.8 ± 83.9	451.8 ± 96.6
	NaCl	84.4 ± 22.6	172.4 ± 20.6	168.4 ± 49.1
Nitrate (µmol mg protein^–1^)	Control	14.9 ± 3.6	28.9 ± 5.1	29.2 ± 4.1
	NaCl	10.2 ± 2.0	25.1 ± 4.7	21.0 ± 3.9*

Means ±SE of three individual experiments are shown.

*Significant difference *P*<0.05 based on ANOVA (Holm–Sidak test) with respect to the corresponding Col-0.

### Arginine bioavailability drives the *cuao8*-dependent phenotype

Several lines of evidences argue for the existence of an arginine-dependent NO production pathway in higher plants similar to that which exists in animals, where arginine is converted into citrulline and NO (Gas *et al.*, 2009; Del Rio, 2011; Corpas and Barroso, 2014). Arginine bioavailability in plants is controlled notably by arginase, which exists under two isoforms in *A. thaliana*, namely ARGH1 and ARGH2, the second counting for ~85% of the total arginase activity (Winter *et al.*, 2015). Interestingly, the manipulation of expression of arginase was previously correlated with modifications of NO production, signaling, and plant stress resistance, possibly via the arginine-dependent NO production pathway (Flores *et al.*, 2008; Shi *et al.*, 2013; Meng *et al.*, 2015). To assay whether this pathway could be involved in the decreased NO production measured in *cuao8* mutants, a series of experiments was performed.

**Fig. 8.**
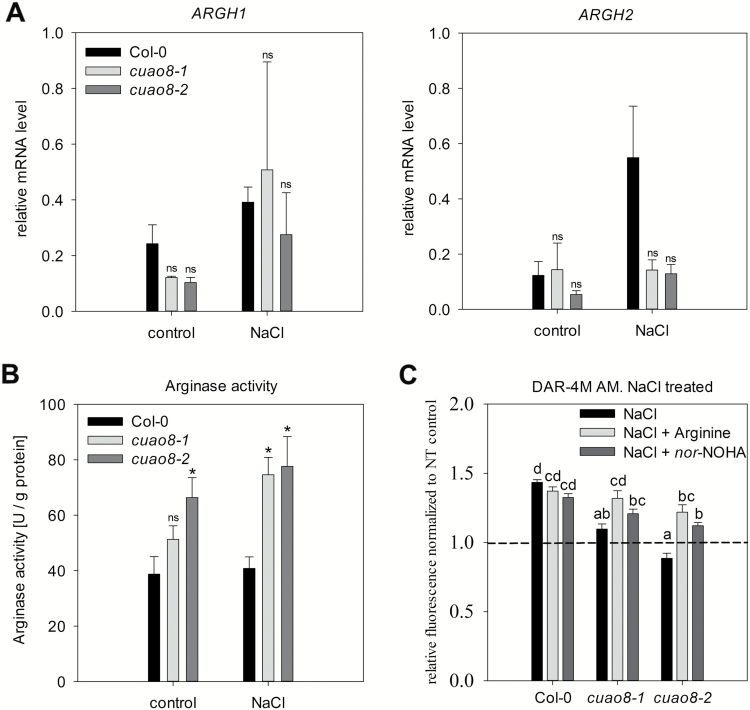
Relative mRNA level of *ARGH1* and *ARGH2*, arginase enzyme activity, and the effect of an arginase inhibitor or arginine on NO production in root tips during NaCl treatment. (A) Relative expression of arginase 1 (*ARGH1*) and 2 (*ARGH2*) in 5-day-old seedlings after control (buffer, 6 h) or NaCl treatment (200 mM, 6 h). ns, non-significant with respect to Col-0. (B) Arginase enzyme activity was measured in total protein extracts of 5-day-old seedlings from Col-0, *cuao8-1*, and *cuao8-2* after control (buffer, 6 h) or NaCl treatment (200 mM, 6 h). The means ±SE of four individual experiments are shown. *Significant differences *P*<0.05 based on ANOVA (Holm–Sidak test) with respect to Col-0. (C) Five-day-old seedlings of Col-0, *cuao8-1*, and *cuao8-2* were stained with DAR-4M AM (5 µM, 1 h) and treated with NaCl (200 mM, 6 h), NaCl plus the arginase inhibitor *nor*-NOHA (100 µM), or NaCl plus arginine (1 mM). The fluorescent signal in the root tips was observed with an epifluorescence microscope. Shown is the relative fluorescence normalized to non-treated controls of each genotype. The means ±SE of at least three individual experiments are shown (*n*=25–48). Different letters indicate a statistically significant difference based on ANOVA on ranks (Dunn’s test. *P*<0.05).

Initially, the expression of *ARGH1* and *ARGH2* was followed in the different lines subjected or not to salt stress (Fig. 8A). Salt stress induced an increase in the expression of both genes in Col-0 seedlings, more pronounced for *ARGH2*, in accordance with previously published data (Shi *et al.*, 2013). A different pattern was observed in the mutant lines, where no significant change for *ARGH2* expression was measured after salt stress.

To confirm this observation further, the total arginase activity from crude extract of Col-0, *cuao8-1*, and *cuao8-2* lines was determined in seedlings exposed or not to salt stress (Fig. 8B). The two insertion lines displayed a strongly and significantly increased arginase activity as compared with Col-0 after salt stress. These results imply a link between CuAO8 and arginase activity in *A. thaliana* during salt stress response.

We then verified whether this link could explain the impaired NO production observed in the *cuao8* mutant lines. We performed NO production tests (DAR-4M AM staining) during salt stress in root tips of seedlings co-treated with the specific reversible arginase inhibitor *nor*-NOHA (Custot *et al.*, 1997) or supplemented with arginine (Fig. 8C). Interestingly, *nor*-NOHA, as well as arginine, could, at least partially, significantly restore the NaCl-induced NO production in both mutant lines. Furthermore, none of these compounds impacted significantly the salt-induced NO production in Col-0. These data confirm the implication of arginase and arginine in CuAO8-dependent NO production during salt stress.

**Fig. 9.**
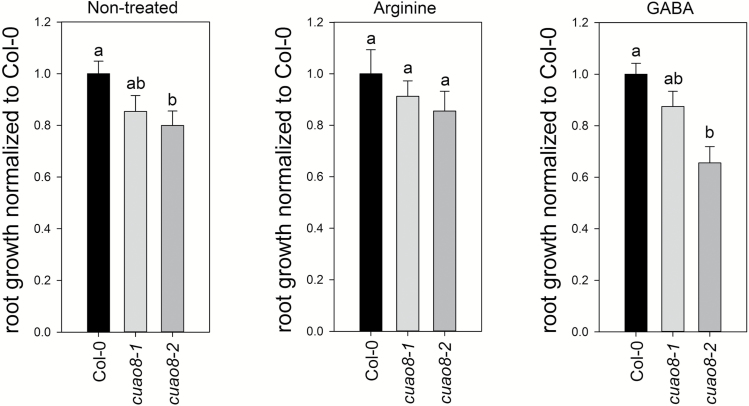
Relative root growth of Col-0, *cuao8-1*, and *cuao8-2* after 11 d of growth on vertical 1/2 MS plates or 1/2 MS plates supplemented with GABA (1 mM) or arginine (1 mM). The means ±SE of at least three individual experiments are shown (*n*=14–22), normalized to Col-0. Different letters indicate a statistically significant difference based on ANOVA on ranks (Dunn’s test. *P*<0.05).

Based on these results, we examined the effect of arginine on the *cuao8*-dependent reduction of the primary root length (Fig. 9). For this purpose, seedlings were grown on 1/2 MS medium supplemented with arginine. Given that GABA synthesis may result from a product generated by the CuAO enzymatic activity (Seiler, 2004), we also included a GABA-negative control to confirm the causality between CuAO8 activity itself and the phenotype. GABA treatment could not restore the normal primary root growth, in contrast to arginine supplementation. This demonstrates that the mutant phenotype is due to the consumption of the substrate rather than the generation of the product from CuAO8 activity since the supplementation with arginine significantly restored the primary root length up to Col-0 levels. Taken together, these results show that the mutant phenotype observed results from a change in arginine bioavailability due to an elevated arginase activity caused by the knockout of CuAO8.

## Discussion

**Fig. 10.**
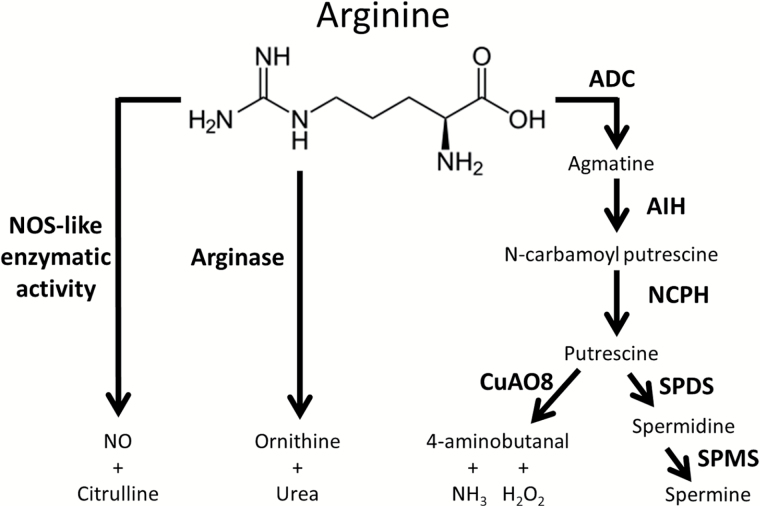
Schematic representation of the arginine metabolic pathway in *A. thaliana*. Arginine is a common substrate for the NOS-like activity, arginase activity, and polyamine synthesis pathway in *A. thaliana*. ADC, arginine decarboxylases; AIH, agmatine iminohydrolase; NCPH, *N*-carbamoyl putrescine hydrolase; NOS, nitric oxide synthase; SPDS, spermidine synthases; SPMS, spermine synthase.

Deciphering the complex mechanisms responsible for the NO production in higher plants is a challenging issue of great interest for a better understanding of the role of NO in plant physiology. NR, putative NOS, electron transport, and putative polyamine oxidases have been suggested as sources of NO in plants (for reviews, see Moreau *et al.*, 2010; Frohlich and Durner, 2011). We were able to demonstrate that the knockout of CuAO8 led to a decreased NO production due to a higher arginase activity (Fig. 8B). Furthermore, reduced primary root growth in *cuao8-1* and *cuao8-2* was correlated to lower NO levels and could be rescued by arginine supplementation. These results point to a regulatory effect of CuAO8 on arginine-dependent NO production during stress responses (Figs 2 , 3) but also during primary root growth (Figs 4, 10).

As PAs are able to induce NO production in root seedlings (Scheler *et al.*, 2013) and the *cuao1* mutant displayed an impaired NO production in response to ABA or PA treatment (Wimalasekera *et al.*, 2011), we sought to determine the NO production in mutant lines for each CuAO predicted gene of *A. thaliana* (Planas-Portell *et al.*, 2013) using seedlings submitted to an elicitor treatment (Fig. 1A). The screen of *A. thaliana* T-DNA insertion lines revealed a reduced NO production to a similar extent in *noa1* and half of the CuAO insertional lines (*atao1*, *cuao1*, *cuao6*, *cuao8*, and *cuao9*). This tendency, even if not significant, correlates with the impairment of several *CuAO* mutant lines in NO synthesis, which reinforces the existing link between PA metabolism and NO production in plants (Tun *et al.*, 2006; Wimalasekera *et al.*, 2011; Scheler *et al.*, 2013; Yang *et al.*, 2014). In our screening system, *cuao8-1* displayed a strong and significant reduction of NO production. To confirm and strengthen this initial observation, we obtained two independent *cuao8* insertion lines, knocked out at the transcript and protein level (Fig. 1B, C), and validated the impaired NO production after elicitor (INA) treatment (Floryszak-Wieczorek *et al.*, 2012; Fig. 2). Because the use of DAF-FM DA for NO detection has been the subject of debate particularly in plant biology (Rumer *et al.*, 2012), we also assayed the impairment of NO production with the Cu_2_(FL2E) probe. This recent tool is much more specific for NO than DAF-FM DA and insensitive to other reactive oxygen or reactive nitrogen species present in the medium (McQuade and Lippard, 2010). The data were confirmed with this second probe, validating the use of DAF-FM DA in our experiments. For the screening experiment, a rather high concentration of INA was applied to provoke a fast and strong NO response. The treatment was afterwards substituted by NaCl to analyze NO production in Col-0, *cuao8-1*, and *cuao8-2* seedlings in a more physiological context. NaCl was chosen since PAs and NO are clearly connected in promoting salt stress resistance in *A. thaliana* (Quinet *et al.*, 2010; Tanou *et al.*, 2014). Almost no NO production was detected in seedling root tips of *cuao8-1* and *cuao8-2* after salt stress, again using two different NO dyes (Fig. 3). In addition to the NO impairment observed after INA treatment, these results demonstrate an involvement of CuAO8 in the general NO production in seedlings.

NO is a signaling compound involved in a wide range of developmental processes in plants, notably controlling primary root growth (Fernandez-Marcos *et al.*, 2011; Liu *et al.*, 2015). The reduced NO production is involved in the observed reduced primary root growth in *cuao8-1* and *cuao8-2* since NO donors could at least partially rescue the root growth phenotype (Fig. 4). This supports the connection of CuAO8 and NO production also in a developmental process, independent of an elicitor or stress treatment.

However, the molecular mechanism linking the knockout of CuAO8 to a reduced NO production is ambiguous. The characterization of the CuAO8 enzymatic activity *in vitro* revealed a typical CuAO activity (Fig. 5B), displaying the highest activity with putrescine as a substrate, as previously described for other CuAOs (Cona *et al.*, 2006; Planas-Portell *et al.*, 2013). Since NO-producing activity was not found in recombinant CuAO8 (data not shown), the effect of CuAO8 on NO formation was more likely to be indirect.

In plants, nitrite reduction is described to be one of the major sources of NO (Meyer *et al.*, 2005; Stöhr *et al.*, 2001; Rockel *et al.*, 2002; Bethke *et al.*, 2004; Igamberdiev *et al.*, 2010; Jeandroz *et al.*, 2016). Interestingly, it has been reported that putrescine is able to reduce the NR activity (Athwal and Huber, 2002; Rosales *et al.*, 2012), and we measured an increased putrescine content in *cuao8-1* and *cuao8-2* (Table 1). In these plants, a decreased *NIA1/NIA2* gene expression was found as compared with Col-0, correlated with a decrease in the potential total NR activity. However, no differences in the actual NR activity between the mutants and Col-0 were measured (Kaiser and Huber, 2001; Rockel *et al.*, 2002; Heidari *et al.*, 2011) which was further confirmed by the similar nitrite/nitrate contents in the three genotypes (Table 2). Together, these results suggest that the CuAO8-dependent NO production in the seedlings is not related to the reductive NO-producing pathway.

In addition to nitrite reduction, an arginine-dependent NO production is also described in plants, similar to the NOS activity described in the animal field. Even though the putative NOS enzymes involved in this pathway remain to be identified, several lines of evidence established the correlation of a consumption of l-arginine with the production of NO in plants (Gas *et al.*, 2009; Del Rio, 2011; Corpas and Barroso, 2014). Arginine is not only a potential substrate for a NOS-like activity in plants but it is also a precursor of PA, the substrate of CuAO (Moschou *et al.*, 2012; Fig. 10). It should be noted that the measurement of proteinogenic amino acids in Col-0, *cuao8-1*, and *cuao8-2* revealed lower arginine contents in *cuao8-1* and *cuao8-2* (Supplementary Fig. S1). Reduced arginine levels corresponding to a reduced NO production in mammalian and plant literature is often correlated with higher arginase activity (Gobert *et al.*, 2000; Li *et al.*, 2001; Flores *et al.*, 2008; Shi *et al.*, 2013; Meng *et al.*, 2015). Arginases metabolize arginine to ornithine and urea, and the reduction of the available arginine pool is one of the main regulatory modes of some NOS activity in mammals (Berkowitz *et al.*, 2003; Ljubisavljevic *et al.*, 2014). Interestingly, a higher arginase activity was measured in *cuao8-1* and *cuao8-2*, and the inhibition of arginases partially recovered the impaired NO production in root tips after salt stress (Fig. 8B, C). Moreover, supplementation with arginine restored the NO production in the mutant lines to almost the Col-0 level and could also reverse the reduced primary root growth phenotype observed for *cuao8-1* and *cuao8-2* (Fig. 9). The higher arginase activity found in *cuao8-1* and *cuao8-2* seems to result from a post-transcriptional regulation as no changes in gene expression of *ARGH1/ARGH2* could be measured in the mutants (Fig. 8A). Moreover, a direct modulation of arginase activity by CuAO8 is unlikely, since both arginase isoforms are localized in the mitochondria (Flores *et al.*, 2008) and a GFP–CuAO8 construct localized in the cytosol and at the plasma membrane (Supplementary Fig. S2). However, nothing is known about a post-translational regulation of *A. thaliana* arginases so far which could reveal a possible regulatory pathway associated with the function of CuAO8 in PA metabolism. Taken together, our work demonstrates that the influence of CuAO8 on arginase activity affects the arginine bioavailability, which has an impact on the putative NOS-like NO production pathway.

Summarizing, this work characterizes for the first time the copper amine oxidase CuAO8 from *A. thaliana*. Insertional mutant lines for this enzyme displayed an impaired NO production in root tips of seedlings submitted to an elicitor treatment or to exposure to salt stress. The *cuao8* mutants displayed an NO-dependent shorter primary root length phenotype. The recombinant protein presented a typical CuAO activity, metabolizing PA and producing H_2_O_2_, with a higher affinity for putrescine. We could demonstrate that the impairment of NO production in these mutants was not caused by a change in the NR activity. However, a higher arginase activity was detected. The resulting modulation of the bioavailability of arginine affected the NO formation probably via a NOS-like NO production route. The impact of CuAO8, and possibly also other CuAOs, on NO production constitutes a new regulatory level of NO signaling in plants during stress response and developmental processes.

## Supplementary data

Supplementary data are available at *JXB* online.

Fig. S1. Metabolic network between polyamines and amino acids, and influence of NaCl treatment on the amino acid levels in Col-0, *cuao8-1*, and *cuao8-2.*

Fig. S2. Subcellular localization of transiently expressed GFP–CuAO8 in *N. benthamiana*.

Table S1. List of PCR primers.

Table S2. List of T-DNA insertion lines.

Table S3. Precursor, product ions, collision energy, and cone voltage of underivatized amino acids and d_5_-Phe for LC-ESI-MS-MS.

## Supplementary Material

supplementary_figures_S1_S2_table_S1_S3

## Abbreviations:

AG: aminoguanidine
ARGH: arginase gene
atao1: *aldehyde oxidase 1*
CaM: calmodulin
cPTIO: 2-(4-carboxyphenyl)-4,4,5,5-tetramethylimidazoline-1-oxyl-3-oxide
CuAO: copper amine oxidase
DAF-FM DA: 4-amino-5-methylamino-2',7'-difluorofluorescein diacetate
DAR-4M AM: diaminorhodamine
DCF-DA: 2′,7′-dichlorofluorescein diacetate
GABA: γ-aminobutyric acid
GSNO: *S*-nitrosoglutathione
INA: 2,6-dichloroisonicotinic acid
MS: Murashige and Skoog
NIA: nitrate reductase gene
Ni-NR activity: nitrite reductase activity
NO: nitric oxide
noa1: *nitric oxide associated 1*
*nor*-NOHA: *N* ω-hydroxy-nor-arginine
NOS: nitric oxide synthase
NR: nitrate reductase
PA: polyamine
SNAP: *S*-nitroso-*N*-acetylpenicillamine.
