# Supplementary material for: Copper amine oxidase 8 regulates arginine-dependent nitric oxide production in Arabidopsis thaliana
Source: J Exp Bot. 2017 Apr 5;68(9):2149–62. doi: 10.1093/jxb/erx105 (PMC5447880; doi:10.1093/jxb/erx105)
Supplement: supplementary_figures_S1_S2_table_S1_S3 [file erx105_suppl_supplementary_figures_S1_S2_table_S1_S3.pdf]

Supplementary data

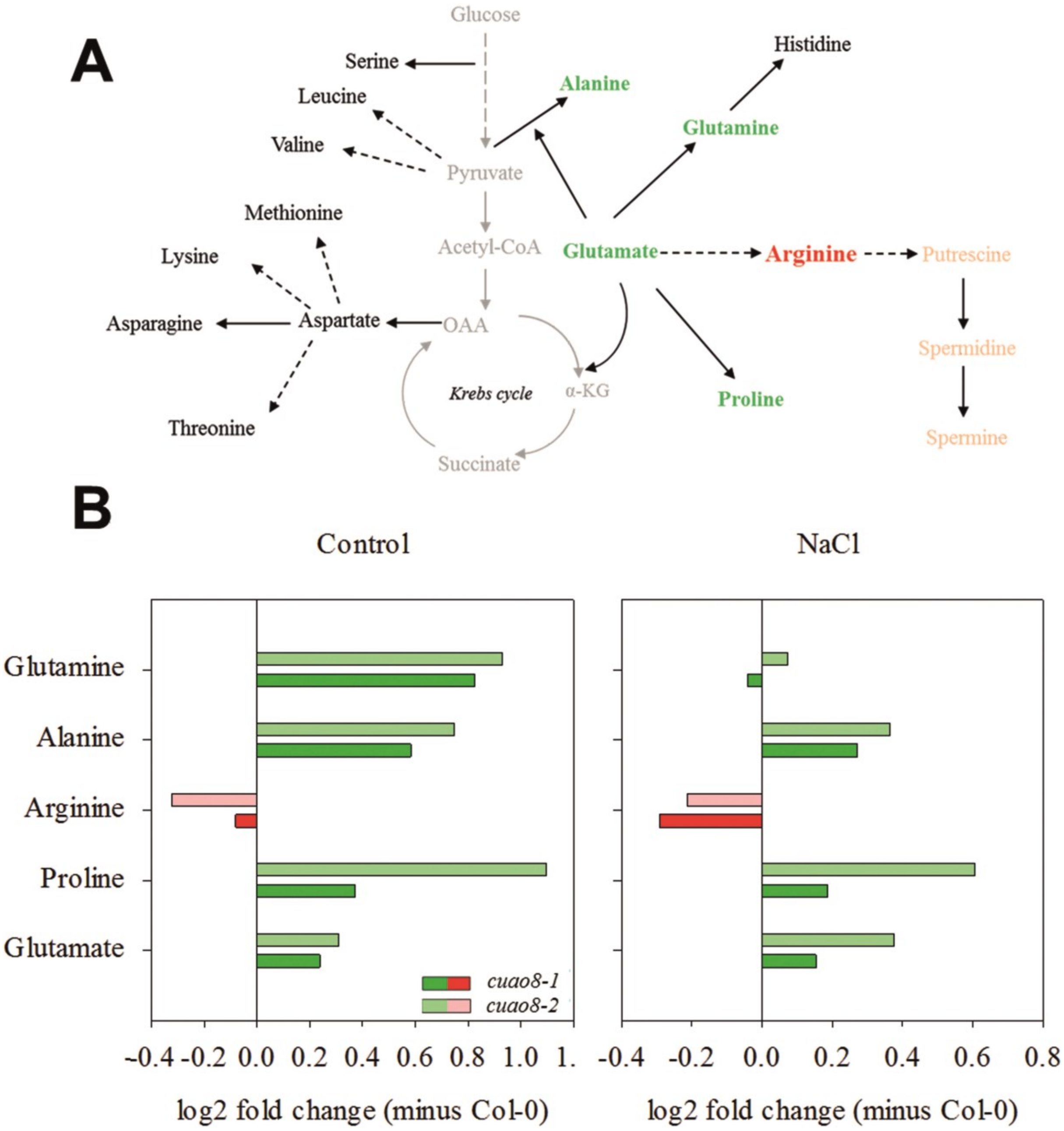

Supplemental Figure 1: Overview of the metabolic network between polyamines and amino acids and the influence of NaCl treatment on the amino acid levels in Col-0, *cuao8-1 and cuao8-2.* (A) Schematic representation of metabolic relationship among polyamines and amino acids (modified from (Mohapatra *et al.*, 2010)). The amino acids written in green/red color display the amino acids with the closest connection to the polyamine pathway. Dashed arrow: metabolic steps in between are left out (B) The log2 fold change of amino acids (shown in (A) in green/red) in five day old seedlings. Amino acids were measured with LC-MS/MS after NaCl (200 mM, 6h) respectively control treatment (buffer; 6h). Means of five individual experiments are presented.

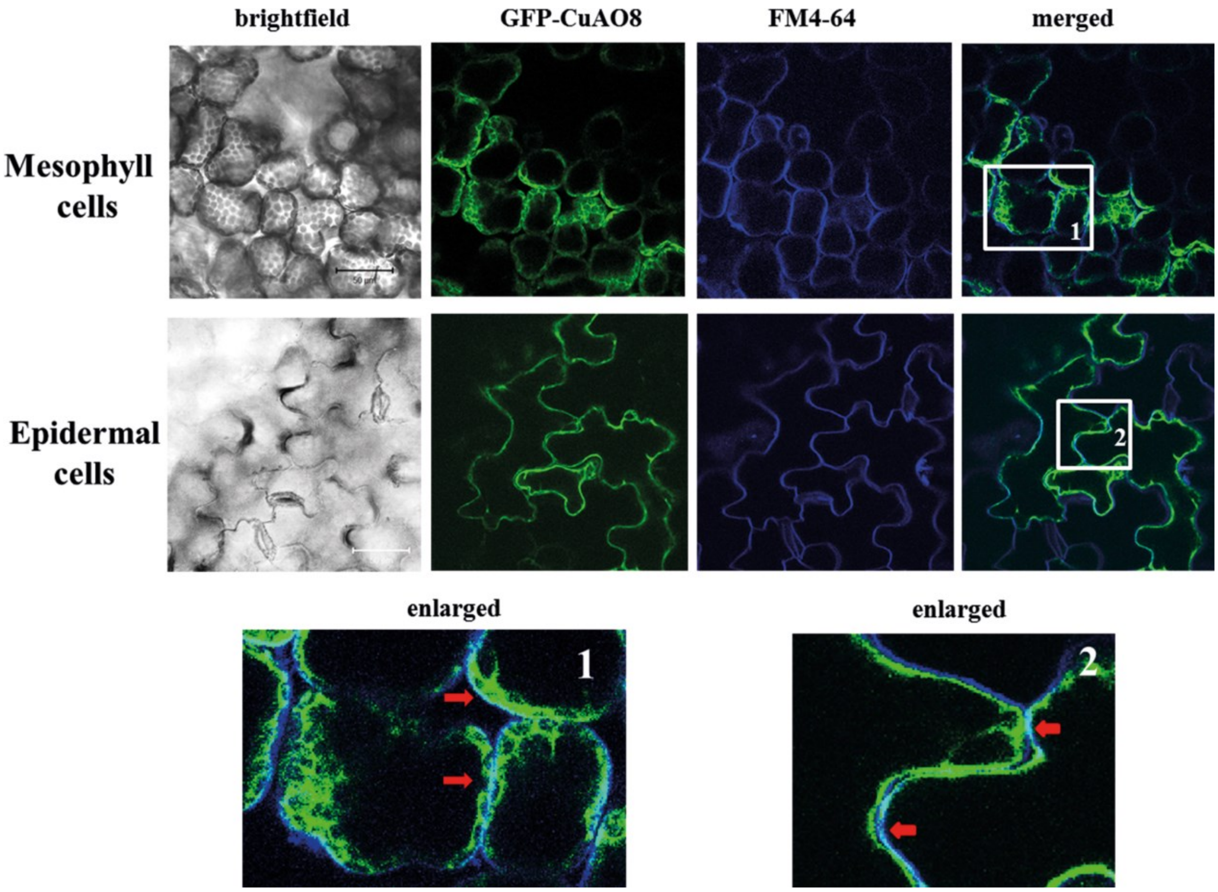

Supplemental Figure 2: Subcellular localization of GFP-CuAO8 in mesophyll and epidermal cells of *N. benthamiana*. GFP-CuAO8 was transiently expressed in five week old *N. benthamiana* cells for five days. The fluorescence was observed with laser confocal microscopy and localized in the cytosol and to a lesser extent at the plasma membrane. FM4-64 (20 µM; 15 min) staining was applied to visualize the plasma membrane. Scale: 50 µm. Light blue color represents overlap between GFP and FM4-64 signal (see red arrow).

Supplemental Table 1: List of PCR primers used in this study.

		Forward primer (5' -> 3')	Reverse primer (5' -> 3')
qPCR primers	*CuAO8*	CATTAGCCGTATGGTCCCAAAGAG	TTGGTTGGCCGAAGTTCAAAGC
	ARGH1	TGAAGCTGGTGATGGAAGAGGAAC	TCCTCCAAGTTTCTCCGAAACAGC;
	ARGH2	CACGGGTTCTAACTGATGTTGGG	AACGGACGCAATGGTTCCTC
	NIA1	CTGAGCTGGCAAATTCCGAAGC	TGCGTGACCAGGTGTTGTAATC;
	NIA2	AACTCGCCGACGAAGAAGGTTG	GGGTTGTGAAAGCGTTGATGGG
	reference gene *UBQ5*	GGTGCTAAGAAGAGGAAGAAG	CTCCTTCTTTCTGGTAAACGT
	reference gene *S16*	TCTGGTAACGAGAACGAGCAC	TTTACGCCATCCGTCAGAGTAT
	reference gene *Tub9*	GTACCTTGAAGCTTGCTAATCCTA	GTTCTGGACGTTCATCATCTGTTC.
Expression	CuAO8-His_6	AAAAAACCATGGCTCAAGTTCACTTAACCATT	AAAAAACTGCAGTTAATGGTGATGGTGATGGTGGCCACTTCCGCTGCCTTCGTTCTTCGTAGTACACTTTGG
Localization	GFP-CuAO8	CACCATGGCTCAAGTTCACTTAACCATTT	TTATTCGTTCTTCGTAGTACACTTTGG

Supplemental Table 2: List of T-DNA insertion lines used for the initial screen.

**Plant line**	**Plant species**	**Eco type**	**SALK-Institute identifier**	**gene**	**AGI code**
cuao1	*Arabidopsis thaliana*	Columbia	SALK_206657	*CuAO1*	AT1G62810
cuao2	*Arabidopsis thaliana*	Columbia	SALK_012167	*CuAO2*	AT1G31710
cuao3	*Arabidopsis thaliana*	Columbia	SALK_095214	*CuAO3*	AT2G42490
cuao4	*Arabidopsis thaliana*	Columbia	SALK_012213	*CuAO4*	AT1G31670
cuao5	*Arabidopsis thaliana*	Columbia	SALK_097684	*CuAO5*	AT3G43670
cuao6	*Arabidopsis thaliana*	Columbia	SALK_124509	*CuAO6*	AT4G12270
cuao7	*Arabidopsis thaliana*	Columbia	SALK_021559	*CuAO7*	AT4G12280
cuao8-1	*Arabidopsis thaliana*	Columbia	SALK_037584	*CuAO8*	AT1G31690
cuao8-2	*Arabidopsis thaliana*	Columbia	SALK_201804	*CuAO8*	AT1G31690
cuao9	*Arabidopsis thaliana*	Columbia	SALK_039444	*CuAO9*	AT4G12290
atao1	*Arabidopsis thaliana*	Columbia	SALK_082394	*AtAO1*	AT4G14940

Supplemental Table 3: Precursor and product ions for LC-ESI-MS-MS analysis of 19 underivatized amino acids and d_5-Phe (internal standard) with their optimized values for collision energy and cone voltage

Compound	Precursor ion $[M+H]^+$ (*m/z*)	Product ion (*m/z*)	Collision energy [eV]	Cone [V]
**Asp**	134	74	14	**20**
**Glu**	148	84	14	**2**
**Ser**	106	60	10	**2**
**Asn**	133	87	10	**66**
**Gln**	147	84	14	**2**
**Tyr**	182	136	14	**6**
**Gly**	76	48	10	**20**
**Pro**	116	70	14	**32**
**Ala**	90	44	6	**20**
**Met**	150	104	10	**2**
**Val**	118	72	10	**24**
**Thr**	120	74	12	**4**
**Phe**	166	120	12	**6**
**d_5-Phe**	168	122	12	**6**
**Leu**	132	86	10	**26**
**Ile**	132	86	10	**26**
**Trp**	205	188	10	**26**
**His**	156	110	14	**32**
**Lys**	147	84	14	**2**
**Arg**	**175**	**70**	**18**	**38**
